# Cell-surface signatures of immune dysfunction risk-stratify critically ill patients: INFECT study

**DOI:** 10.1007/s00134-018-5247-0

**Published:** 2018-06-07

**Authors:** Andrew Conway Morris, Deepankar Datta, Manu Shankar-Hari, Jacqueline Stephen, Christopher J. Weir, Jillian Rennie, Jean Antonelli, Anthony Bateman, Noel Warner, Kevin Judge, Jim Keenan, Alice Wang, Tony Burpee, K. Alun Brown, Sion M. Lewis, Tracey Mare, Alistair I. Roy, Gillian Hulme, Ian Dimmick, Adriano G. Rossi, A. John Simpson, Timothy S. Walsh

**Affiliations:** 1 University Division of Anaesthesia, Department of Medicine, Addenbrooke’s Hospital, University of Cambridge, Box 93, Hills Road, Cambridge, CB2 0QQ England UK; 2 MRC Centre for Inflammation Research, University of Edinburgh, 47 Little France Crescent, Edinburgh, Scotland UK; 3 Edinburgh Critical Care Research Group, University of Edinburgh School of Clinical Sciences, Edinburgh, Scotland UK; 4 Intensive Care Unit, Guy’s and St Thomas’ Hospital NHS Foundation Trust, London, England UK; 5 Edinburgh Clinical Trials Unit, Usher Institute of Population Health Sciences and Informatics, University of Edinburgh, Edinburgh, Scotland UK; 6 Intensive Care Unit, Western General Hospital, Crewe Road South, Edinburgh, Scotland UK; 7 BD Biosciences, San Jose, CA USA; 8 IncellDx, Menlo Park, CA USA; 9 Applied Cytometry, Sheffield, England UK; 10 Vascular Immunology Research Laboratory, Rayne Institute (King’s College London), St Thomas’ Hospital, London, England UK; 11 Integrated Critical Care Unit, Sunderland Royal Hospital, Sunderland, England UK; 12 Flow Cytometry Core Facility Laboratory, Faculty of Medical Sciences, Centre for Life, Newcastle University, Newcastle, England UK; 13 Institute of Cellular Medicine, Newcastle University, Newcastle upon Tyne, England UK

**Keywords:** Cross infection, Immunoparesis, Immunophenotyping, Monocytes, Neutrophils, T-lymphocytes, regulatory

## Abstract

**Purpose:**

Cellular immune dysfunctions, which are common in intensive care patients, predict a number of significant complications. In order to effectively target treatments, clinically applicable measures need to be developed to detect dysfunction. The objective was to confirm the ability of cellular markers associated with immune dysfunction to stratify risk of secondary infection in critically ill patients.

**Methods:**

Multi-centre, prospective observational cohort study of critically ill patients in four UK intensive care units. Serial blood samples were taken, and three cell surface markers associated with immune cell dysfunction [neutrophil CD88, monocyte human leucocyte antigen-DR (HLA-DR) and percentage of regulatory T cells (T_regs_)] were assayed on-site using standardized flow cytometric measures. Patients were followed up for the development of secondary infections.

**Results:**

A total of 148 patients were recruited, with data available from 138. Reduced neutrophil CD88, reduced monocyte HLA-DR and elevated proportions of T_regs_ were all associated with subsequent development of infection with odds ratios (95% CI) of 2.18 (1.00–4.74), 3.44 (1.58–7.47) and 2.41 (1.14–5.11), respectively. Burden of immune dysfunction predicted a progressive increase in risk of infection, from 14% for patients with no dysfunction to 59% for patients with dysfunction of all three markers. The tests failed to risk stratify patients shortly after ICU admission but were effective between days 3 and 9.

**Conclusions:**

This study confirms our previous findings that three cell surface markers can predict risk of subsequent secondary infection, demonstrates the feasibility of standardized multisite flow cytometry and presents a tool which can be used to target future immunomodulatory therapies.

**Trial registration:**

The study was registered with clinicaltrials.gov (NCT02186522).

**Electronic supplementary material:**

The online version of this article (10.1007/s00134-018-5247-0) contains supplementary material, which is available to authorized users.

## Take home message

**Table:** 

ΓÇÿSecondary infections are a major concern in intensive care and have been convincingly associated with immune dysfunction occurring as a result of critical illnesses. Effective targeting of immunomodulatory therapies requires the ability to identify patients who are at risk, and this study presents three immune cell markers which additively predict the risk of subsequent secondary infectionΓÇÖ	

## Introduction

Critical illness occurs when a sterile [1, 2] or infective [3] insult leads to organ dysfunction [4]. A hallmark of critical illness is systemic inflammation and immune-mediated organ damage [5]. However it is increasingly clear that this immune activation is accompanied by an equally pronounced immunodepression [6], and that a persistence of this immune duality is associated with a complicated intensive care unit (ICU) course [7]. If effective treatments for this complex state of immune dysfunction are to be developed, we need to identify clinical and laboratory markers for it.

A significant complication in intensive care is the development of nosocomial infection, which occurs in 20–40% of patients [8]. The development of such infections is relevant to patients and the healthcare system, as they are associated with increased morbidity, mortality, prolonged need for organ support, and greater overall illness costs [8]. ICU-acquired infections are also a major driver of antibiotic use, which contributes to increased antimicrobial resistance and the risk of drug toxicity [9]. We, and others, have previously demonstrated a strong association between immune dysfunction and the subsequent development of nosocomial infection [1, 3].

We previously published the findings of a single-centre study examining three markers associated with immune dysfunction [3], namely neutrophil CD88 (as a marker of C5a-mediated neutrophil dysfunction [10, 11]), monocyte human leucocyte antigen-DR (HLA-DR) (as a marker of monocyte deactivation [12, 13]) and the proportion of regulatory T cells (T_regs_, associated with an increased risk of secondary infection [14]). These three markers independently, and additively, predicted the subsequent development of nosocomial infection [3].

The results of single-centre studies can be hard to replicate, but development of clinically useable measures of immune function to guide trials of immunomodulatory therapy in critical illness [15] requires accurate, reproducible immunophenotyping tools. The aim of the ImmuNe FailurE in Critical Therapy study was to confirm our previously identified markers associated with immune dysfunction, and their ability to identify risk of subsequent nosocomial infection in a multicentre study, using standardized flow cytometry. Some of the results of these studies have been previously reported in the form of an abstract [16].

## Methods

The study protocol has been published [17]. The study was approved by National Health Service Research Ethics Committees and was performed in accordance with the ethical standards laid down in the 1964 Declaration of Helsinki and its later amendments.

### Setting and participants

Patients were recruited from the four participating ICUs, based in the UK. Details of the recruiting units are shown in the supplemental section (Table S1). Inclusion criteria were adult patients admitted to ICU and receiving either invasive mechanical ventilation or two or more other organ systems support, who were predicted to remain in ICU for at least 48 h [17]. Written, informed assent was obtained from the nearest relative or personal consultee, and retrospective consent was sought from all patients who regained capacity. A full list of exclusion criteria is in the supplemental section.

### Sampling schedule

Tripotassium ethylenediaminetetraacetate (K3-EDTA) anticoagulated blood samples were taken on the day of study enrolment, and then at 2-day intervals until day 12 with patients being followed for determination of infection to day 16. Patients discharged to hospital wards remained in the study. Patients discharged home during the study period were followed up using hospital records to ascertain re-admission with infection.

### Definition of infection

We used methods developed in our previous study to identify infection [3, 17]. Briefly, researchers blinded to the immune phenotyping data assessed patients for the development of infections which were defined using the Hospitals in Europe Link for Infection Control through Surveillance criteria [18] (see supplemental methods). Suspected infections that did not meet the criteria for confirmed infection underwent independent, blinded review by two expert intensive care clinicians (see supplemental methods). The onset of infection was defined as the time the relevant microbiology sample was taken. Only the first infection was defined as the outcome of interest.

### Flow cytometric standardization and sample staining

All sites used the same flow cytometry analyser, the FACS Canto II (BD Biosciences, San Jose, CA, USA), and antibodies from the same batch, using study standard operating procedures. Machines were standardized by monthly matching of target values using a common batch of Cytometry Setup and Tracking (CS&T) beads (BDB), and daily internal quality control using the same common batch of CS&T beads. Details of the fluorophores and antibodies are reported in Table S2.

### Immunophenotyping measures

The primary measures were those used in the published study [3], namely surface neutrophil CD88 (nCD88, as a marker associated with C5a-mediated neutrophil dysfunction [10, 11]), monocyte HLA-DR (mHLA-DR, as a marker associated with monocyte deactivation [19]) and the proportion of CD4 T cells expressing a regulatory phenotype (CD4^+^/CD25^++^/CD127^−^ T_regs_) [3, 20]. These were compared against simple enumeration of leucocyte populations and lymphocyte to neutrophil ratios which have previously been reported to be predictive of subsequent infection [21, 22].

A study of the reliability and reproducibility of the measures of cell surface markers was conducted as set out in the published protocol [17] (see supplemental section).

### Defining cut-off for ‘dysfunction’

Changes in antibody clones, fluorophores and flow cytometers necessitated by the multicentre standardization process meant we were unable to directly compare the marker values from the current study and the previous study [3]. Therefore, we re-derived optimum cut-off values for cell-surface markers using receiver operating characteristic (ROC) curve analysis, defining ‘dysfunction’ and ‘no dysfunction’ using the same method [3]. Briefly, patients were categorized using values from the sample taken most proximally to the relevant clinical end-point (death, infection or discharge without infection). For patients who acquired an infection, the sample used was the one taken closest to but preceding the 48-h time point before the infection. This was chosen to identify markers that changed prior to infection developing.

### Statistical analysis

Statistical analysis followed the statistical analysis plan that was finalized prior to database lock (see supplemental section). The sample size calculation is set out in the protocol paper [17]. Assuming a 35% nosocomial infection rate [3], to estimate a 50% positive predictive value with 95% confidence interval width of 39–61% would require 200 patients. Optimum cut-offs were defined by constructing ROC curves comparing values from samples 48 h before an infection with samples from patients who did not develop infection, using Youden’s index [23]. Post hoc analyses to adjust for clinical and demographic predictors of infection were undertaken; these were not prespecified in the analysis plan but were included in response to independent review.

### Modelling potential clinical use

In a pre-planned evaluation of the clinical potential for these markers to alter decision-making [17], we examined the ability of the immune dysfunction measurements to predict subsequent infection, ICU length of stay and duration of organ support. We used the optimum cut-offs from ROC analysis and calculated their predictive ability when measured at baseline, and days 2–4, 6–8 and 10–12 post study enrolment. We chose these time points as clinically relevant times at which the immune phenotyping might be undertaken and operationalised in routine care. We modelled predictive performance with a two-step process: first, using the best-performing test (monocyte HLA-DR) to select patients with low HLA-DR; and second, then classifying patients with either low neutrophil CD88 or elevated T_regs_ (or both) as ‘highest risk’.

## Results

### Recruitment and development of infection

We recruited 148 patients. Time and budgetary restraints limited recruitment of the predefined numbers. Supplemental Fig. S1 shows the recruitment diagram. A total of 138 patients were available for analysis. Patients were enrolled a median 1.5 days (IQR 0.7–2 days) after admission to ICU. Demographic and clinical data are shown in Table 1.

**Table 1:** Clinical and demographic features of patients who did and did not develop secondary infection following admission to intensive care

Parameter	Infection (51)	No infection (87)
Age (years), median (IQR)	66 (48–74)	65 (53–74)
Male gender, *n* (%)	32 (63%)	55 (63%)
Mean (SD) functional co-morbidity index score	2.0 (2.2)	1.8 (1.6)
Smoking status		
Current	15 (29%)	21 (24%)
Ex-smoker	11 (22%)	19 (22%)
Non-smoker	18 (35%)	27 (31%)
Unknown	7 (14%)	20 (23%)
Admission reason (some patients fall into both ‘sepsis’ and one other category)		
Sepsis	15 (29%)	40 (46%)
Surgery	16 (31%)	16 (18%)
Trauma	5 (10%)	2 (2%)
Other	19 (37%)	39 (45%)
APACHE II score, mean (SD)	14.6 (6.7)	15.4 (6.3)
SOFA score, mean (SD)	4.8 (2.5)	5.5 (3.1)
Admission white cell count/mm^3^, median (IQR)	12,900 (9100–16,800)	11,950 (7900–14,250)
Admission neutrophil count/mm^3^, median (IQR)	10,360 (7000–14,140)	9200 (7000–12,200)
Admission lymphocyte count/mm^3^, median (IQR)	1140 (750–1780)	1000 (620–1500)
Arterial line	51 (100%)	87 (100%)
CVC	48 (94%)	78 (89%)
Endotracheal tube	51 (100%)	86 (99%)
Enteral/parenteral nutrition	47 (93%)/9 (18%)	82 (94%)/8 (9%)
Urinary catheter	51 (100%)	85 (98%)
Corticosteroids (< 400 mg hydrocortisone-equivalent/24 h)	18 (35%)	37 (43%)
Stress ulcer prophylaxis	49 (96%)	80 (92%)
Antibiotics in 72 h prior and/or within 24 h of ICU admission	41 (80%)	79 (91%)
ICU length of stay in days, median (IQR)	15 (10–24)	7 (5–14)
Mortality		
ICU	7 (14%)	20 (23%)
Hospital	18 (35%)	26 (30%)
Infection related	12 (67%)	10 (39%)

*IQR* interquartile range, *SD* standard deviation, *APACHE* acute physiology and chronic health evaluation, *SOFA* sequential organ failure assessment, *CVC* central venous catheter

In total 51 (37%) patients developed secondary infections after study entry; 34 (67%) met the criteria for confirmed infection whilst 17 (33%) were deemed “highly likely” on expert review. Infection sites, organisms and relationship to primary infection are summarised in supplemental Table S3. Infection occurred a median 7 days (IQR 3–11) after ICU admission. In nine patients, infection developed within 48 h of study entry; these patients were excluded from the ROC analysis, but included in a sensitivity analysis.

### Association of markers of immune dysfunction with subsequent infection

The three tests all demonstrated high intra- and inter-rater reliability (see supplemental results section).

Reduced nCD88, reduced mHLA-DR and elevated proportions of T_regs_ were all associated with subsequent development of infection when applying optimal ROC cut-offs (Table 2); odds ratios (95% CI) were 2.18 (1.00–4.74), 3.44 (1.58–7.47) and 2.41 (1.14–5.11), respectively. Area under the ROC curve for each marker is reported in the supplemental section (Table S4). None of the clinical or demographic variables were significant independent predictors of subsequent infection. Adjustment for potential confounding by these variables did not result in significant changes to the unadjusted odds ratios (Table S5). Values plotted for each marker dichotomised by group (did versus did not develop infection) on the different days before infection events occurred showed that changes in CD88 and HLA-DR tended to occur 2–3 days prior to the events (supplemental results Fig. S2a, b).

**Table 2:** Predictive performance of markers at the optimal cut-offs defined by ROC analysis

Marker	Cut-off	Spec	Sens	NPV	PPV	OR
CD88	≤ 9609	0.49 (0.39–0.60)	0.69 (0.53–0.82)	0.77 (0.64–0.87)	0.39 (0.29–0.52)	2.18 (1.00–4.74)
Monocyte HLA-DR	≤ 2009	0.63 (0.52–0.73	0.67 (0.51–0.8)	0.80 (0.68–0.88)	0.47 (0.34–0.60)	3.44 (1.58–7.47)
T_regs_ as % of CD4 cells	≥ 12.12	0.64 (0.53–0.74)	0.57(0.41–0.72)	0.76 (0.64–0.85)	0.44 (0.30–0.58)	2.41 (1.14–5.11)

Numbers are point estimate (95% CI). CD88 and monocyte HLA-DR are expressed in arbitrary fluorescence units

*Spec* specificity, *Sens* sensitivity, *NPV* negative predictive value, *PPV* positive predictive value, *OR* odds ratio

### Marker performance in survival analysis

**Fig. 1:**
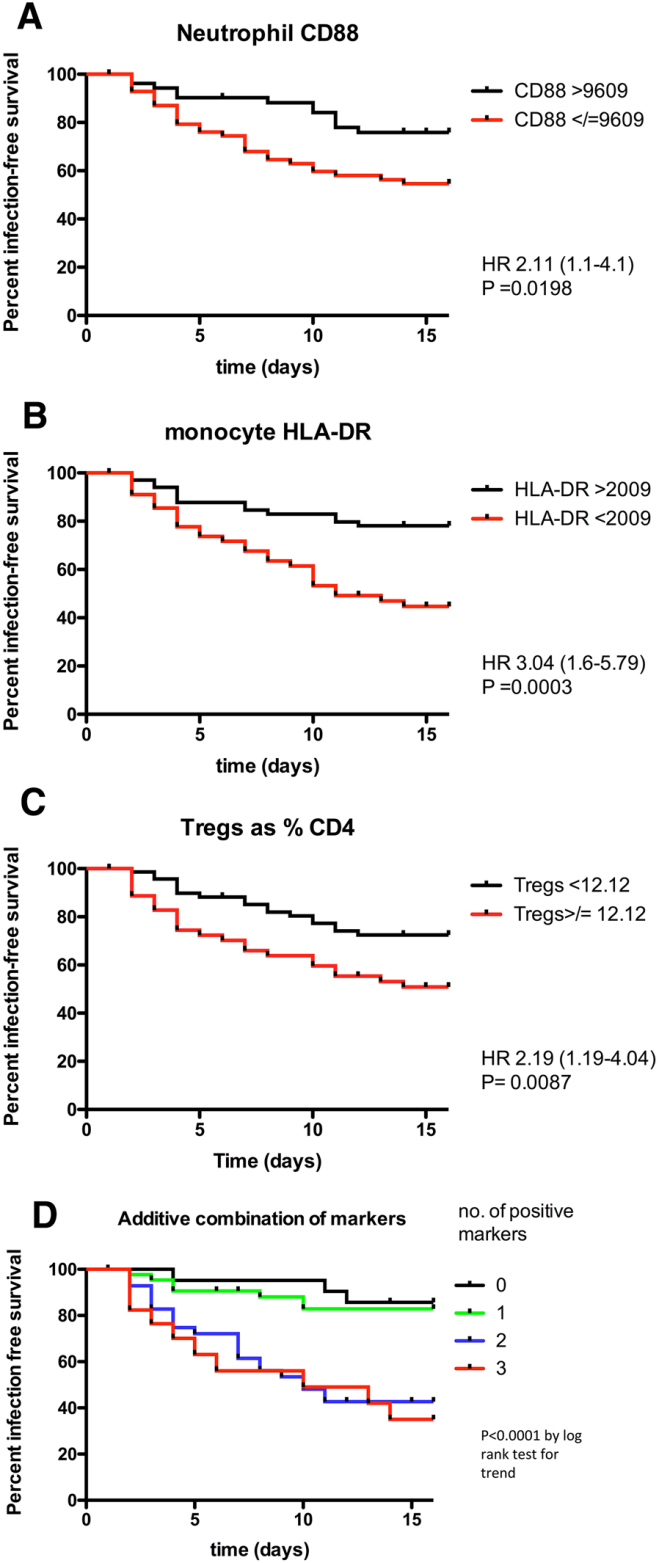
Survival curves for patients dichotomised by markers at the cut-offs shown. **a** Neutrophil CD88 expression, **b** total monocyte HLA-DR expression, **c** T_regs_ as a  percentage of all CD4^+^ cells. **d** Additive combination of markers *p* value by log-rank test (panels **a**–**c**) and log-rank test for trend (panel **d**). Hazard ratios for combined markers are shown in Table 3

In survival analysis, all three measures showed a significant association with the hazard of infection (Fig. 1a–c). As for the odds ratios, adjustment for clinical/demographic variables did not result in significant changes to the hazard ratios (see Table S5).

Baseline marker values did not predict subsequent infection, with or without adjustment for clinical variables (supplemental Table S6).

### Additional and sensitivity analyses

Sensitivity analyses that included patients who developed secondary infection within the 48-h window of first blood sampling are shown in the supplement (Table S7). The relationships between the markers and other clinical outcomes, namely length of stay, days alive and free of organ support, and SOFA scores are shown in the supplement (Tables S8, S9).

We found no predictive value for total leucocyte or differential counts (supplemental Table S10).

### Marker combinations for predicting subsequent infection

Using the best cut-offs derived from the ROC analysis for each of the three biomarkers, we assigned each ‘dysfunction’ a score of 1 (no dysfunction scored 0). The number of dysfunctions was summed to give a cumulative total (range 0–3). With this approach, an increase in the number of immune dysfunctions was associated with a progressive increase in the risk of subsequent infection (Table 3, Fig. 1d). When the combined markers were used, the area under ROC curve (AUC ROC) was 0.72 (95% CI 0.62–0.81); this was larger than each individual biomarker AUC (range 0.57–0.64; Table S4). Adjustment for potential clinical confounders did not significantly alter this relationship (Table S11).

**Table 3:** Predictive performance of additive combination of the three markers

Number of dysfunctions	*n*	Infections	95% CI for proportion	OR (95% CI)	Adjusted OR	HR	Adjusted HR
0	21	3 (14%)	(5–35%)	Indicator	Indicator	Indicator	Indicator
1	45	7 (16%)	(8–29%)	1.1 (0.25–4.7)	1.00 (0.22–4.41)	1.53 (0.41–5.64)	1.20 (0.30–4.49)
2	46	22 (48%)	(34–62%)	5.5 (1.4–21.3)	5.10 (1.30–19.99)	6.04 (1.83–20.00)	5.19 (1.55–17.42)
3	17	10 (59%)	(36–78%)	8.5 (1.8–40.7)	8.31 (1.70–40.71)	6.00 (1.65–21.90)	6.62 (1.81–24.21)

Analysis of odds ratio (OR) by logistic regression and hazard ratios (HR) from Cox proportional hazards model. Adjusted analysis reports results adjusted for SOFA score, comorbidity and use of steroids

### Modelling of clinical use

The modelling of potential clinical use of these tests, which would most likely be to guide immunomodulatory therapy, aimed to dichotomise patients into particularly ‘low’ risk (unlikely to benefit from immunomodulation) and ‘high’ risk (immunomodulation potentially could modify risk) for subsequent infection.

When the clinical modelling strategy set out in the “Methods” was used, the sample taken at study baseline demonstrated no significant ability to predict subsequent infection (see Table 4, Figure S3a), similar to the analysis of single markers at baseline (Table S6). In sensitivity analyses that selected higher sensitivity thresholds for ‘low’ monocyte HLA-DR, there was no improvement in the predictive ability of the test at baseline (data not shown).

**Table 4:** Dichotomisation of patients using the proposed test criteria, with proportions and hazard ratio for developing infection subsequent to the test

Study day	*N* of patients assigned to high risk/low risk categories	*N* (%) infections (high risk/low risk) *p* value by Fisher’s exact test	Hazard ratio for development of infection (95% CI) *p* value by log-rank test	Median (IQR) ICU LOS (high risk/low risk) *p* value by Mann–Whitney	Median (IQR) study days alive without organ support (high risk/low risk) *p* value by Mann–Whitney
0 (enrolment)	65/71	26 (40%)	1.18 (0.67–2.1)	12 (8–18)	6 (0–11)
		25 (35%)		12 (6–20)	7 (0–12)
		*p* = 0.60	*p* = 0.55	*p* = 0.89	*p* = 0.86
2–4	47/75	20 (43%)	2.80 (1.40–5.70)	16 (12–21)	5 (0–9)
		14 (19%)		11 (7–18)	9 (0–12)
		*p* = 0.007	*p* = 0.005	*p* = 0.008	*p* = 0.02
6–8	35/57	14 (40%)	4.30 (1.70–10.20)	17 (13–23)	5 (0–9)
		7(12%)		14 (10–21)	8 (0–11)
		*p* = 0.004	*p* = 0.002	*p* = 0.13	*p* = 0.12
10–12	21/38	3 (14%)	2.10 (0.40–11.80)	20 (14–31)	0 (0–7)
		3 (8%)	*p* = 0.40	16 (13–23)	6 (0–10)
		*p* = 0.66		*p* = 0.07	*p* = 0.04

ICU length of stay and duration of organ support-free study days is shown for patients by category at each time point

*LOS* length of stay, in days

In contrast, the sample taken between study days 2 and 4 had potentially useful discriminant value for patients at low and high risk of subsequent secondary infection (see Table 4, Fig. S3b). ‘High-risk’ patients also had lower infection-free survival on survival analysis [HR 2.8 (95% CI 1.4–5.7) *p* = 0.005 by log-rank test], and significantly longer ICU length of stay and duration of organ support (Table 4). This predictive ability was also found at days 6–8. A similar, but non-statistically significant, pattern was also found at days 10–12 (Table 4). Adjustment for potential clinical confounders did not significantly alter the predictive ability of these markers at any of the time points examined (Table S12). Selecting higher sensitivity thresholds for ‘low’ monocyte HLA-DR increased the overall sensitivity of the test but this occurred at the expense of specificity (data not shown).

In a hypothetical model of enriching a trial population with ‘high-risk’ patients for an immunomodulatory intervention, we found the numbers of patients needed to treat (and consequently the size of any trial) could likely be substantially reduced using this precision-medicine approach (see Tables S13, S14, supplemental section). For example, if this strategy was used to enrol high-risk patients based on a test carried out on ICU day 3–5 post admission, a 30% relative risk reduction in subsequent infection (80% power) could be detected with a sample size of 436, compared to 806 if ‘all comers’ were included.

## Discussion

This is the first report of multicentre, multiparametric immunophenotyping for the determination of immune dysfunction amongst critically ill adults and one of only two studies [3] to examine multiple markers over time. Through this study we have confirmed the role of three markers associated with leucocyte dysfunction in predicting the development of nosocomial infection. Although each marker alone had relatively modest predictive ability, in combination they had stronger association with the risk of infection. These results are strikingly similar to our previous findings providing confirmatory evidence [3].

The demographic and clinical data (Table 1) demonstrate that the patients were broadly similar at the time of study enrolment regardless of whether they subsequently develop an infection, and had similar severity of illness scores and requirement for organ support on ICU admission. Adjustment for these factors did not alter the predictive ability of the cell-surface markers (see Tables S5, S11–12). These findings contrast with other studies which found that age and illness severity are strong predictors of subsequent nosocomial infection [24]. This discrepancy may be explained by differences between the cohorts of patients recruited. We deliberately recruited a group of patients who were already at high risk of secondary infection, confirmed by the 37% incidence observed. The markers we present have also been associated with severity of illness [25–27], and therefore their use will already encode similar information to that contained in severity scores. We contend that in the severely ill patient cohort we studied, clinical and demographic features are unlikely to reliably discriminate risk of subsequent infection.

We found that measuring immune dysfunction with the baseline sample (taken a median 1.5 days after ICU admission) shows poor predictive ability for subsequent infections. By contrast, samples taken between study days 2 and 8 (median 3–9 days after ICU admission) had better predictive ability for subsequent infections, and were also associated with longer ICU stay and greater need for organ support. The median time to infection from a positive test on the day 2–4 sample was 5 days, which appears a plausible interval in which an immunostimulatory therapy could work [28]. Using this approach would allow targeting of immunomodulatory therapies to patients who might have greatest risk–benefit balance from these therapies especially through reduction in secondary infections. This might also translate into reduced length of stay and infection-associated morbidity and mortality [28]. The impact of nosocomial infection on mortality is relatively limited [24], but the costs of prolonged hospital stay and organ support are significant and could be significantly substantially impacted on by targeted immunotherapy. A precision medicine approach has greatest chance of cost-effectiveness given the potential high cost of these therapies.

It is interesting to note that patients who developed secondary infections demonstrated an impaired recovery in both monocyte HLA-DR and neutrophil CD88 relative to those who did not develop infections (Fig. S2), suggesting that persistence of dysfunction is an important risk factor for subsequent infection, similar to an association reported in trauma patients [1]. Whether immunomodulatory therapies should be started at ICU admission and modified at days 3–5, or not started until days 3–5 will need to be determined by future randomized controlled trials.

Our study has a number of strengths. By using common protocols and reagents, the same model of flow cytometry analyser and cross-site standardization, we have been able to obtain comparable results from geographically and clinically diverse units. We have also been able to explore a number of putative markers of immune dysfunction, validating two, which we had established previously alongside the benchmark monocyte HLA-DR.

We used rigorous criteria for the determination of infection, and expert consensus for those suspected infections that did not meet the criteria. Sensitivity analyses demonstrated considerable overlap in the 95% confidence intervals for the predictive performance of the markers, either when early (< 48 h after enrolment, Table S7) or only confirmed infections were included (data not shown).

The fields of immunophenotyping and critical illness-induced immune dysfunction are relatively new, and as such there are no established benchmarks or levels for defining ‘normal’ and ‘abnormal’ measures. Although this study used development of infection to define cut-offs for immune dysfunction, further external validation of markers at these specific cut-offs is required. At present the assays need a high degree of machine standardization and further work is required to extend their usability to different flow cytometers or towards a near-patient test. The extension of the existing QuantiBRITE system (BD Biosciences, San Jose, CA) used for monocyte HLA-DR quantification to other fluorophores and other cell surface markers would be one potential solution to this issue.

Whilst the tests presented have relatively modest performance against typical ‘diagnostic’ tests, they are not designed to diagnose secondary infection, but rather to stratify the risk of developing secondary infection. As such the markers show comparable performance to risk markers used in other acute illnesses, such as predictors of mortality in acute exacerbations of chronic obstructive pulmonary disease [29] or major cardiac events in unstable angina [30]. Monocyte HLA-DR is in current use for risk stratification in clinical trials (NCT02361528), and we demonstrate in this study that its predictive ability is significantly enhanced by the addition of the two additional markers. As we demonstrate in modelling potential use (Tables S13, 14), the use of these markers could substantially alter the conduct of trials of immunomodulatory therapies, thus increasing the likelihood of successful trials and potentially increasing the cost-effectiveness of such novel therapies.

In conclusion, our study confirms our previous findings of the ability of neutrophil CD88, monocyte HLA-DR and the percentage of T_regs_ to predict secondary infection in patients requiring intensive care and organ support, and provides novel data on the time course of dysfunction and clinical relevance of the optimum timing of sampling in predicting risk of infection. The combination of markers allows for a more nuanced assessment of infection risk, with important implications for developing stratified medicine tools to support future immunomodulatory therapies amongst this group of patients.

## Electronic supplementary material

This article has an online data supplement.

Below is the link to the electronic supplementary material.

Supplementary material 1 (DOCX 63 kb)
