# Supplementary material for: Cell-surface signatures of immune dysfunction risk-stratify critically ill patients: INFECT study
Source: Intensive Care Med. 2018 Jun 7;44(5):627–35. doi: 10.1007/s00134-018-5247-0 (PMC6006236; doi:10.1007/s00134-018-5247-0)
Supplement: Supplementary file 1 — Supplementary material 1 (DOCX 63 kb) [file 134_2018_5247_MOESM1_ESM.docx]

**Cell surface signatures of immune dysfunction risk stratify critically ill patients: INFECT Study**.

**Supplemental section**.

**Supplemental methods**

**Ethical review and registration**

| **Unit name/**  **characteristic** | **Royal Infirmary of Edinburgh** | **St Thomas’s Hospital, London** | **Sunderland Royal Hospital** | **Western General Hospital, Edinburgh** |
| --- | --- | --- | --- | --- |
| **Type** | Liver/  General | General | General | Neuroscience/  General |
| **Beds** | 28 | 48 | 16 | 16 |
| **Admissions/ year** | 2400 | 1200 | 936 | 630 |
| **Open/Closed*** | Closed | Closed | Closed | Closed |

Ethical approvals were granted by the Scotland A Research Ethics Committee (13/SS/0022) for Scottish sites and Warwick and the Coventry Research Ethics Committee (13/WM/0207) for English sites. The study was registered with clinicaltrials.gov (NCT02186522).

**Settings and participants**

Details of the participating units are summarized in Table S1 below.

**Table S1: Characteristics of participating units.**

All units had standard infection control practices including audited hand washing, bedside aprons, ventilator-bundle, vascular line bundle, routine infection surveillance and antibiotic guidelines. * ‘Closed’ units indicate that the primary treatment responsibility lies with intensive care physicians.

**Inclusion and exclusion criteria**

Study inclusion and exclusion criteria were as follows:

Inclusion criteria:

1. Adult patients (>16 years old (Scotland) or >18 years old (England))
2. Receiving support of level 3 care (i.e. requiring invasive support of respiratory system alone, or two or more other organ systems (hemofiltration, inotropes/vasopressors)
3. Predicted to remain in ICU for at least 48 hours.

Exclusion criteria:

1. Patients not expected to survive beyond 24 hours
2. Known or suspected ICU-acquired infection at time of screening (healthcare-acquired infection acquired outside ICU was not an exclusion)
3. Known inborn errors of immune function
4. Immunosuppression on ICU admission or in the 24 hours prior to ICU admission (corticosteroids up to 400mg hydrocortisone equivalent daily dose was permitted)
5. HIV, hepatitis B, hepatitis C infection
6. Extra-corporeal membrane oxygenation (ECMO)
7. Pregnancy
8. Previous enrolment in the study.

**Definitions of infection**

Any new infection occurring after 48 hours of ICU admission was deemed ‘ICU-acquired’. For consistency infections arising within 48 hours of ICU discharge were also deemed ‘ICU-acquired’.

Infections were defined prior to the start of the study as follows, based on the Hospitals in Europe Link for Infection Control through Surveillance (HELICS) criteria^E1^

***a) Ventilator-associated pneumonia (VAP)*:**  Requires radiographic, clinical and microbiological criteria to be met:

i. Radiological criteria.

Chest X-ray (CXR) or computed tomography (CT) scan showing new infiltrates, or worsening infiltrates without evidence of pulmonary edema, and either pyrexia of >38^o^C or white cell count (WCC) in peripheral blood >12000/mm^3^ or <4000/mm^3^.

These must be combined with one or more clinical criteria.

ii. Clinical criteria.

Worsening oxygenation – any increase in FiO_2_ to maintain PaO_2_ target, or an increase in positive end expiratory pressure (PEEP), frequency or tidal volume, proning or paralysis to facilitate ventilation

OR

Relevant clinical chest findings – auscultatory finding of crepitations, crackles or decreased air entry

OR

Increased/changed sputum – any increase in volume, presence of muco-purulent or muco-purulent-bloody sputum.

Combined with

iii. Microbiological criteria.

The above radiological and clinical criteria must be combined with positive quantitative culture at >10^4^ colony forming units (CFU) per milliliter (ml) of bronchoalveolar lavage (BAL) fluid (or >10^3^ CFU/ml on protected specimen brush (PSB) sampling) or positive culture of pleural fluid or positive culture from a pulmonary/pleural abscess.

Where the diagnosis of VAP was suggested by mini-BAL/endotracheal aspirate, or where growth was below the 10^4^ CFU/ml threshold or without any positive microbiology, adjudication was required.

***b) Hospital-acquired pneumonia (HAP*)**

i.e. nosocomial pneumonia in non-mechanically ventilated patients (or patient in ICU for >48 hours but ventilated for <48 hours), required the same fulfillment of criteria as for VAP except that sputum cultures with heavy growth of a single organism constituted a confirmed infection.

***c) Catheter-associated infections***

Positive culture (where semi-quantitative cultures were available >15 CFU) from an indwelling vascular line combined with either

Local inflammation and pus (catheter-related infection (CRI)) or

Improvement of inflammatory markers within 48 hours of removal (CRI) or

Culture of the same organism from a peripheral blood culture (catheter-related blood stream infection (CRBSI).

***d) Blood stream infection***

One positive culture of a typical pathogen, coupled with evidence of systemic inflammation (WCC >12,000/mm^3^ or <4000/mm^3^, temperature ≥ 38^o^C).

***e) Urinary tract infection***

Growth of 2 or fewer organisms at ≥ 10^5^ CFU/ml combined with evidence of systemic inflammation (WCC >12,000/mm^3^ or <4000/mm^3^, temperature >38^o^C or shock without another identifiable cause).

**f*) Soft-tissue or surgical site infection***

Evidence of pus/inflammation at site of presumed infection combined with a positive culture.

**g) *Infective diarrhoea***

Evidence of diarrhea (3 or more loose stools in 24 hour period or use of a fecal collector) combined with culture positive for a diarrheal organism or detection of an enteropathogenic toxin.

**h) *Intra-abdominal infection***

Evidence of intra-abdominal collection identified on surgical or radiological investigation, combined with positive culture from surgical specimen, needle aspirate or drain.

**i) *Spontaneous bacterial peritonitis (SBP)***

Evidence of infected ascites (fluid with >250 neutrophils/mm^3^, or abdominal pain and blood WCC >12,000/mm^3^ or <4000/mm^3^, temperature ≥ 38^o^C in the presence of ascites without other source of infection) combined with positive ascitic fluid culture. Negative ascitic fluid culture in the presence of clinical criteria for SBP would be ‘probable’ infection.

**j) *Sinusitis***

Evidence of facial sinus fluid collection on radiological grounds (plain film, CT or ultrasound) or direct clinical observation of purulent discharge from a sinus combined with positive culture of fluid.

Infecting organisms could be bacterial, fungal or viral. Viral infections had to be accompanied by clinical, radiological or histological evidence of tissue inflammation (e.g. herpes simplex stomatitis, Varicella pneumonitis, cytomegalovirus colitis). Viral positivity without evidence of tissue inflammation was classified as reactivation.

**A ‘confirmed’** infection was one that met the above criteria.

**Independent review of suspected, non-confirmed infections**

Where an infection was suspected by the clinical team but did not meet the criteria above, the case details were sent to members of an expert consensus panel who were blinded to the immune cell data. The case was reviewed by two members of the panel who independently adjudicated the infection as either ‘probable’ or ‘unlikely’. If the two clinician judgments were different, a third panel member was asked to review the case with the majority decision being recorded. The panel consisted of five of the investigators (ACM, MH, AR, AB and TSW) who are all senior ICU clinicians with at least 10 years post-graduate experience in Intensive Care Medicine. Each reviewed cases from units in which they were not practicing.

A **‘highly likely’** infection was assigned when the panel clinicians thought there was, on the balance of probabilities, an infection present and would consider antibiotic treatment and/or source control if the patient’s clinical condition merited it. This category could include positive microbial cultures. An example would be culture of a classically non-pathological organism (e.g. single cultures of coagulase negative cocci or diphtheroids) associated with clinical evidence of infection/systemic inflammation. Classically non-pathological organisms could be classified as ‘confirmed’ infections where there was strong evidence

Infection was considered **‘unlikely’** when the panel clinician thought there was a low probability of infection and would not consider antibiotic treatment and/or source control. Although positive microbial cultures could be included in this, this would be culture of a classically non-pathological organism (e.g. single cultures of coagulase negative cocci or diphtheroids) without evidence of systemic inflammation/infection or mixed growth of commensal organisms.

Systemic evidence of infection required the presence of systemic inflammatory response syndrome (SIRS), defined as 2 or more of the following: heart rate >90 beats per minute, WCC >12000/mm^3^ or <4000/mm^3^ or >10% band types, respiratory rate >20 breaths per minute or mechanical ventilation, and temperature of >38°C or <36°C. Additional evidence considered included reports of large numbers of neutrophils on sample microscopy, and clinical examination findings of pus or inflamed tissue.

‘Unlikely’ infection combined with a positive microbial culture would constitute **colonization**.

**Flow cytometric standardization and sample staining**

Leukocyte cell surface staining was conducted using antibodies supplied by BD Biosciences. All sites used antibodies from the same batch (see Table S2 for details of antibody-fluorophore combinations used). Staining, data capture and storage were conducted in accordance with the study standard operating procedures. Briefly, blood samples (collected in Tri-potassium ethylenediamine tetraacetate (K3-EDTA)) were taken and transported on ice to the laboratory. Analysis was completed within 4 hours of phlebotomy. 50μl of blood was mixed with the relevant antibodies and incubated for 30minutes at 4^o^C, before red cell lysis using PharmLyse (BD Biosciences), washing twice and resuspending with Hanks’ Balanced Salt Solution (HBSS). Tubes were kept at 4^o^C and analyzed immediately.

Analysis was conducted using VenturiOne (Applied Cytometry, Sheffield, UK).

| **Marker** | **Clone** | **Fluorophore** |
| --- | --- | --- |
| CD15 | W6D3 | FITC |
| CD88 | C85-4124 | PE |
| CD14 | MΦP9 | APC-H7 |
| HLA-DR | G46-6 | APC |
| CD4 | SK3 | APC-H7 |
| CD25 | MA251 | PerCP-Cy5.5 |
| CD127 | HIL-7R-M21 | PE-Cy7 |

**Table S2:** **Fluorophore:antibody clone combinations used in the study**

CD, cluster of differentiation; HLA, human leukocyte antigen; FITC, fluorescein isothiocyanate; PE, phycoerythrin; APC, allophycocyanin; PerCP, peridinin chlorophyll protein; Cy, cyanine.

**Set up of FACS Canto II machine.**

Initial compensations for multi-color panels were undertaken using compensation beads (BD Biosciences) and, when compensation beads failed to bind antibody, blood.  The compensated template specific for each antibody panel and instrument was used for all subsequent acquisitions unless instrument variation was detected, at which point the compensation matrix would again be verified.  All sites used the same flow cytometer FACS Canto II (BD Biosciences) and were set up using monthly matching of Cytometry Setup and Tracking (CS&T) target values to the highest mid bead value. This avoided high optical background within the analysis range on any instrument within the group. Site matching was also undertaken following any adjustment or replacement of an optical, laser or detection component.  Daily internal quality control was undertaken to ensure that there were no significant daily variations in machine performance. A common lot of CS&T was used for both these processes.

**Reliability study**

Studies were conducted to ensure the flow cytometric readings of each marker were reproducible. Inter- and intra-observer reliability studies were conducted with three expert readers of the data. Fifty anonymized data files were distributed to the three readers and read to determine inter-observer agreement, with 13 anonymized files sent subsequently for intra-observer agreement. After a preliminary reliability study, an optimization stage was carried out with the experts meeting to further improve flow cytometric gating strategies in problematic markers. A second reliability study was then conducted using the same sample size, with the results presented as inter- and intra-rater correlation coefficients.

A single reader read each of the final data files from the study.

**Statistical analysis**

The data analysis followed the statistical analysis plan that was finalized prior to database lock. The primary outcome was the development of ICU-acquired infection within the 16-day study period (defined as confirmed or probable infection).

The main analysis of the primary outcome was of the positive predictive value (PPV) and negative predictive value (NPV) of immune dysfunctions in predicting nosocomial infection, as well as the predictive ability of combinations of immune dysfunction. The primary analysis included both ‘confirmed’ and ‘probable’ infections as ‘infections’, with analysis by ‘confirmed’ infections only conducted as a sensitivity analysis. Sensitivity and specificity were determined. 95% confidence intervals were calculated for all measures of predictive accuracy.

As in the previous study ^E2^, ‘immune function/dysfunction’ was defined by the time point before the first nosocomial infection occurred, censored for 48 hours prior to infection. The cut-off for ‘dysfunction’ was defined using the procedure used in the previous study ^E2^, as set out in the main manuscript.

The relationship between baseline sample value and the development of secondary infection was assessed by univariate logistic regression, followed by adjustment for *a priori*-defined clinical factors associated with development of infection, selected to reflect factors related to pre-existing health status, severity of illness and immunosuppressive treatments ^E3^ (co-morbidity as measured by functional comorbidity index ^E4^, severity of organ failure by Sequential Organ Failure Assessment (SOFA) score ^E5^ and use of corticosteroids below the 400mg hydrocortisone equivalent exclusion).

The ability of the dichotomized measures to predict infection was determined by contingency table analysis, deriving sensitivity, specificity, positive and negative predictive values, and odds ratios (OR). Kaplan-Meier (KM) curves were plotted and analyzed by log-rank test. Hazard ratios were generated from the KM curves. Sensitivity analyses were conducted including patients who developed infection within 48 hours of the initial blood sample being taken and, separately, excluding patients with “highly likely” infections.

The secondary outcome of ‘worst SOFA score’ (defined as peak SOFA score and sum of the worst individual component scores) was analyzed by marker ‘positive/negative’ status, using a normal linear model. The secondary outcome ‘length of stay in ICU’ was analyzed for each marker using a Cox model of time to discharge, with death being considered a censoring event and patients dichotomized into 'immune dysfunction' and 'no dysfunction' as one independent variable and adjusting for functional co-morbidity index (total score), receiving corticosteroids and baseline SOFA score. Days alive and free from organ support were summarized and compared between marker-positive and -negative patients using a normal linear model.

We also undertook additional analyses, not included in the original analysis plan. These were analyses of the predictive ability of the markers measured at the point of dichotomization into 'immune dysfunction' and 'no dysfunction'; and the clinical modelling adjusted for baseline SOFA score, functional co-morbidity index (total score) and use of steroids. We also examined whether the findings were robust to substitution of Acute Physiology and Chronic Health Evaluation (APACHE) II score for SOFA score, and age for functional comorbidity index. In the modelling of clinical use, we additionally adjusted for SOFA score on the day of sampling.

Analysis was performed using SASv9.4 (Cary, NC, USA) and Prism (Graphpad Software, La Jolla, CA, USA). A 5% significance level and 95% confidence intervals (CI) were used in all analyses.

**Supplemental results**

| **Site of infection** | **N (Frequency of all infections)** | **Organisms from this site** |
| --- | --- | --- |
| **Pneumonia**  **(of which Ventilator-associated)** | 35  (VAP 28) | ***Citrobacter koseri, Enterobacter spp, Escherichia coli,* Group B *Streptococcus spp., Haemophilus influenzae, Klebsiella pneumoniae, Moraxella catarrhalis, Proteus mirabilis, Pseudomonas aeruginosa, Serratia marcescens, Stenotrophomonas maltophilia,***  ***Staphylococcus aureus,* Yeasts *(Candida spp.), sterile/no sample*** |
| **Renal/Urinary tract infection** | 15 | ***Enterobacter spp, Enterococcus spp, Escherichia coli, Klebsiella pneumoniae,* Yeasts *(Candida spp.)*** |
| **Surgical site/soft tissue infection** | 9 | ***Coliforms (no further speciation), Enterococcus spp, Escherichia coli, Proteus mirabilis, Pseudomonas aeruginosa, Serratia marcescens, Staphylococcus aureus*** |
| **Blood stream infection (without overt source)** | 7 | ***Klebsiella pneumoniae, Klebsiella oxytoca, Serratia marcescens, Staphylococcus aureus*, other *Staphylococci,* Yeasts *(Candida spp.)*** |
| **Vascular catheter-associated infection** | 6 | ***Citrobacter koseri, Staphylococcus aureus,* other *Staphylococci*** |
| **Sinusitis** | 1 | ***Staphylococcus aureus*** |

**Table S3: sites of infection and organisms grown on culture.**

Some patients developed more than one infection, and some cultures grew more than one organism. Suspected infections without positive culture underwent the adjudication process and were judged to be “highly likely”.

15 patients who had infective diagnoses on admission developed secondary infection, 12 patients developed secondary infections at different sites from the source of their primary infection. Of the 3 patients developing secondary infection at the same site as the primary infection, different organisms were cultured at time of secondary infection (all 3 patients presented with viral pneumonitis and developed bacterial secondary infection a median of 9 (range 7-11) days after presentation). One patient grew the same organism from the same site, having had an apparent clinical cure in between, and this was judged to be persistent primary infection and not recorded as a secondary infection.

**Reliability analysis**

In the reliability analysis, two measures (nCD88 and mHLA-DR) had very high intra- and inter-rater reliability (all intra-class correlation coefficients >/= 0.99). The percentage of T_regs_ had an inter-rater reliability of 0.90, and intra-rater reliability of 0.96 for two readers and 0.77 for the third reader.

**Selecting optimal cut-offs for marker dichotomisation**

The optimal cut-off for marker dichotomisation was undertaken by calculating the Youden index from the relevant ROC curves. The values for the area under ROC curve are shown in Table S4 below.

| **Marker** | **AUC ROC** |
| --- | --- |
| **Neutrophil CD88** | 0.57 |
| **Monocyte HLA-DR** | 0.64 |
| **T_regs_ as % of CD4 cells** | 0.59 |

**Table S4: Area under curve for ROC curves from the three markers**

**Additional and sensitivity analyses**

Additional post-hoc analyses adjusting for potential clinical confounders were undertaken at the request of peer reviewers. Adjustment did not significantly alter the odds ratios or hazard ratios for the markers, with the adjusted point estimates well within the confidence intervals for the unadjusted markers (Table S5). Substitution of SOFA score by APACHE II score and functional co-morbidity index (FCI) by age did not lead to significantly different results (data not shown)

| **Marker** | **Adjusted OR** | **P-value** | **Adjusted HR** | **P-value** |
| --- | --- | --- | --- | --- |
| **CD88**  **(+ve/-ve)**  **FCI**  **Baseline SOFA**  **Steroids (no or yes)** | **2.12 (0.97-4.65)** | **0.06** | **2.02 (1.04-3.89)** | **0.037** |
|  | 1.04 (0.83-1.30) | 0.77 | 1.09 (0.89-1.33) | 0.41 |
|  | 0.90 (0.78-1.03) | 0.13 | 0.902 (0.80-1.01) | 0.09 |
|  | 0.92 (0.35-2.39) | 0.86 | 0.96 (0.45-2.03) | 0.91 |
| **Monocyte HLA-DR**  **(+ve/-ve)**  **FCI**  **Baseline SOFA**  **Steroids** | **3.61 (1.63-7.97)** | **0.0015** | **3.11 (1.63-5.93)** | **0.0006** |
|  | 1.03 (0.82-1.29) | 0.82 | 1.1 (0.91-1.33) | 0.32 |
|  | 0.88 (0.76-1.02) | 0.09 | 0.89 (0.79-1.00) | 0.06 |
|  | 1.01 (0.38-2.70) | 0.99 | 0.91 (0.42-1.98) | 0.82 |
| **T_regs_ as % of CD4 cells**  **(+ve/-ve)**  **FCI**  **Baseline SOFA**  **Steroids** | **2.35 (1.10-5.04)** | **0.029** | **2.24 (1.21-4.16)** | **0.011** |
|  | 1.05 (0.84-1.31) | 0.68 | 1.08 (0.89-1.31) | 0.43 |
|  | 0.90 (0.78-1.04) | 0.16 | 0.89 (0.79-1.01) | 0.06 |
|  | 1.07 (0.41-0.81) | 0.89 | 1.10 (0.51-2.38) | 0.81 |

**Table S5: Adjusted odds ratios (by binary logistic regression) and hazard ratio (by Cox proportional hazards model) for the three markers of immune dysfunction.**

Markers were adjusted for functional comorbidity Index, baseline SOFA score, and use of steroids. Adjustment for days of invasive ventilation did not alter the predictive ability of the markers in this model (data not shown)

| **Marker** | **Odds Ratio (OR) (95% CI) (for 1 SD increase in marker value)** | **p-value** | **Adjusted OR (95% CI)** | **Adjusted p-value** | **Adjusted HR (95% CI)** | **Adjusted p-value (Cox model)** |
| --- | --- | --- | --- | --- | --- | --- |
| **CD88** | 0.79 (0.45,1.40) | 0.42 | 0.79 (0.47,1.34) | 0.39 | 0.84 (0.57,1.26) | 0.40 |
| **mHLA-DR** | 0.79 (0.52,1.19) | 0.26 | 0.77 (0.49,1.21) | 0.25 | 0.81 (0.56,1.17) | 0.26 |
| **% Tregs** | 1.32  (0.93,1.87) | 0.13 | 1.31 (0.91,1.90) | 0.15 | 1.18 (0.93,1.50) | 0.17 |

**Table S6: The ability of a 1 standard deviation change in baseline marker value to predict subsequent infection.**

Columns 2/3 show results from univariate logistic regression, columns 4/5 show results from adjusted logistic regression including the three immune variables and clinical factors (functional comorbidity index, use of steroids and baseline SOFA score). Columns 6/7 show results from Cox proportional hazards models including each of the immune dysfunction markers and clinical factors (functional comorbidity index, use of steroids and baseline SOFA score). Substitution of APACHE II score for baseline SOFA and age for FCI did not significantly change the analysis, and none of the potential clinical confounders achieved statistical significance.

A pre-planned sensitivity analysis, including the 9 patients who developed secondary infection within the 48-hour window of first blood sampling, was undertaken, using the cut-offs derived above. All three markers showed similar point estimates and considerable overlap of 95% confidence intervals for the diagnostic performance measures when the primary analysis (main manuscript Table 2) and sensitivity analysis (Table S7) were compared. In restricting analysis to confirmed infections the absolute cut-offs selected were different, but the diagnostic performance and likelihood ratios were similar (Data not shown).

| **Measure** | **Cut-off** | **Spec** | **Sens** | **NPV** | **PPV** | **OR (95% CI)** | **Hazard ratio** |
| --- | --- | --- | --- | --- | --- | --- | --- |
| **CD88** | ≤ 9609 | 0.49 (0.39-  0.6) | 0.67 (0.52-0.79) | 0.72 (0.59-0.83) | 0.44 (0.32-0.55) | 1.95 (0.95-4.01) | 1.80  (1.02-3.29) |
| **Monocyte HLA-DR** | ≤2009 | 0.63 (0.52-0.73) | 0.63 (0.48-0.76) | 0.74 (0.63-0.84) | 0.50  (0.37-0.64) | 3.00 (1.42-5.92) | 2.45  (1.39-4.34) |
| **T_regs_ as % of CD4 cells** | ≥12.12 | 0.64 (0.53-0.74) | 0.57 (0.42-0.71) | 0.72 (0.61-0.81) | 0.48 (0.35-0.62) | 2.38 (1.17-4.83) | 2.07  (1.19-3.61) |

**Table S7: sensitivity analysis for predictive performance of markers including patients developing infection within 48 hours of first research blood sample.**

CD88 and monocyte HLA-DR are expressed in arbitrary fluorescence units.

Spec, specificity; Sens, sensitivity; NPV negative predictive value; PPV positive predictive value.

T_regs_ were also examined using the threshold determined in the previous study ^E3^ of 9.2%. At this threshold T_regs_ were not significant predictors in either contingency table or-time to-event analysis. The method of identifying T_regs_ differed between the two studies and the proportion of T_regs_ was significantly higher in both those developing and those not developing infection in the current study (data not shown).

Having low mHLA-DR (as defined in the main manuscript) was significantly associated with having fewer ‘days alive and free from organ support’ (median 2 days (IQR 0-9) vs. 9 (IQR 4-12); p=0.001 by Mann-Whitney U test). Elevated T_regs_ (>12.12%) were borderline significant (median 5 (IQR 1-11) vs. median 9 (IQR 1-13); p=0.0508) whilst low nCD88 showed no significant association (median 6 (IQR 1-12) vs. 5.5 (IQR1-11); p=0.79). Low mHLA-DR was significantly associated with ICU length of stay (HR 0.59 (95% CI 0.38-0.90, p=0.01), and remained significant when adjusted for admission SOFA score, FCI and use of steroids. Neither CD88 nor T_regs_ demonstrated such a relationship (see Table S8). None of the measures were significantly associated with maximum organ failure, as measured by peak SOFA or sum of maximum component scores (see Table S9).

| **Measure** | **Cut-off** | **Unadjusted HR (95% CI) for time to discharge from ICU** | **p value** | **Adjusted HR** | **Adjusted p value** |
| --- | --- | --- | --- | --- | --- |
| **CD88** | ≤ 9609 | 0.93 (0.61-1.42) | p=0.73 | 0.94 (0.62-1.44) | p=0.79 |
| **Monocyte HLA-DR** | ≤2009 | 0.59 (0.38-0.90) | p=0.01 | 0.58 (0.38-0.89) | p=0.01 |
| **T_regs_ as % of CD4 cells** | ≥12.12 | 0.84 (0.56-1.28) | p=0.42 | 0.82 (0.54-1.3) | p=0.36 |

**Table S8**: **Cox regression analysis of markers for time to discharge from ICU.**

Hazard Ratio (HR) is for marker positive compared to marker negative. Adjusted HR is from Cox regression analysis adjusting for functional comorbidity index, admission SOFA score and receipt of cortico-steroids.

| **Marker** | **Score** | **Marker negative**  **Mean (95% CI score)** | **Marker positive**  **Mean (95% CI score)** | **p value (linear regression)** |
| --- | --- | --- | --- | --- |
| **CD88** | **Peak SOFA score** | 7.75 (2.19,13.31) | 6.90 (1.95,11.86) | 0.08 |
|  | **Maximum sum of SOFA** | 9.61 (2.33,16.88) | 9.16 (2.07,16.26) | 0.50 |
| **Monocyte HLA-DR** | **Peak SOFA score** | 6.94 (1.46,12.42) | 7.65 (2.69,12.61) | 0.14 |
|  | **Maximum sum of SOFA** | 8.86 (1.57,16.14) | 9.93 (3.04,16.83) | 0.10 |
| **Tregs** | **Peak SOFA score** | 7.23 (1.28,12.77) | 7.33 (1.81,12.85) | 0.84 |
|  | **Maximum sum of SOFA** | 9.27 (2.28,16.26) | 9.47 (2.03,16.91) | 0.76 |

**Table S9: Linear regression analysis of relationship between marker positivity and highest recorded (peak) SOFA and sum of highest recorded SOFA components (maximum sum of SOFA).**

| **Marker** | **Cut-off** | **AUC ROC** | **OR** | **P-value (Fishers exact test)** |
| --- | --- | --- | --- | --- |
| **White cell count** | >8200/mm^3^ | 0.53 | 1.89 (0.70-5.15) | 0.25 |
| **Neutrophil count** | >6480/mm^3^ | 0.52 | 2.40 (0.97-5.73) | 0.07 |
| **Lymphocyte count** | >3070/mm^3^ | 0.52 | 1.35 (0.61-2.99) | 0.54 |
| **Neutrophil:lymphocyte ratio** | <15.47 | 0.52 | 1.63 (0.59-4.52) | 0.46 |

**Table S10: Predictive ability of leucocyte count and leucocyte sub-set derived values for development of subsequent secondary infection.**

| **Number of dysfunctions** | **Adjusted OR** | **P value** | **Adjusted HR** | **P-value** |
| --- | --- | --- | --- | --- |
| **0** | reference | 0.0006 | reference | 0.0002 |
| **1** | 1.00 (0.23-4.4) |  | 1.16 (0.30-4.49) |  |
| **2** | 5.10 (1.3-20.00) |  | 5.19 (1.55-17.43) |  |
| **3** | 8.31 (1.70-40.71) |  | 6.62 (1.81-24.21) |  |
| **FCI** | 1.05 (0.82-1.33) | 0.72 | 1.11 (0.92-1.33) | 0.29 |
| **Baseline SOFA** | 0.89 (0.76-1.04) | 0.14 | 0.89 (0.79-1.01) | 0.07 |
| **Steroids** | 1.00 (0.35-2.81) | 0.99 | 1.00 (0.46-2.19) | 1.0 |

**Table S11: Odds ratios for number of marker dysfunctions by binary logistic regression and hazard ratios by Cox proportional hazards model, adjusted for Functional Comorbidity Index, baseline SOFA score, and use of steroids.** Substitution of SOFA score by APACHE II score and functional co-morbidity index (FCI) by age did not lead to significantly different results (data not shown)

| **Study day** | **Adjusted OR (95% CI)** | **P-value** | **Adjusted HR (95% CI)** | **P-value** |
| --- | --- | --- | --- | --- |
| **0 (enrolment)**  **FCI**  **SOFA score**  **Steroids** | 1.07 (0.52-2.20) | 0.86 | 1.10 (0.63-1.92) | 0.75 |
|  | 1.07 (0.88-1.31) | 0.49 | 0.93 (0.84-1.03) | 0.18 |
|  | 0.91 (0.8-1.04) | 0.19 | 1.16 (0.97-1.39) | 0.10 |
|  | 0.89 (0.41-1.93) | 0.77 | 0.86 (0.47-1.56) | 0.86 |
| **Day 2-4**  **FCI**  **SOFA score**  **Steroids** | **3.90 (1.63-9.27)** | **0.002** | **2.87 (1.41-5.82)** | **0.004** |
|  | 1.04 (0.82-1.31) | 0.75 | 1.08 (0.88-1.33) | 0.46 |
|  | 0.88 (0.74-1.04) | 0.13 | 0.90 (0.78-1.04) | 0.16 |
|  | 0.70 (0.28-1.73) | 0.44 | 0.74 (0.35-1.54) | 0.42 |
| **Day 6-8**  **FCI**  **SOFA score**  **Steroids** | **4.74 (1.62-13.93)** | **0.005** | **3.79 (1.46-9.76)** | **0.006** |
|  | 0.97 (0.73-1.31) | 0.86 | 0.96 (0.75-1.23) | 0.73 |
|  | 0.97 (0.80-1.19) | 0.79 | 0.96 (0.81-1.14) | 0.66 |
|  | 1.23 (0.42-3.59) | 0.71 | 1.28 (0.52-3.15) | 0.60 |
| **Day 10-12**  **FCI**  **SOFA score**  **Steroids** | 2.05 (0.33-12.53) | 0.44 | 1.96 (0.36-10.56) | 0.44 |
|  | 1.00 (0.61-1.66) | 1.0 | 0.97 (0.60-1.56) | 0.89 |
|  | 1.13 (0.79-1.62) | 0.51 | 1.09 (0.78-1.53) | 0.60 |
|  | 0.21 (0.19-2.37) | 0.21 | 0.28 (0.29-2.75) | 0.28 |

**Table S12: For the proposed test criteria (as set out in methods section of main manuscript), odds ratio was calculated by binary logistic regression and hazard ratio from Cox proportional hazards model for development of infection subsequent to the test.** Results are adjusted for functional co-morbidity index (FCI), SOFA score on day of sampling, and use of steroids. Substitution of SOFA score by APACHE II score and functional co-morbidity index (FCI) by age did not lead to significantly different results (data not shown).

| **Relative risk reduction (prevalence of subsequent infection)** | **0.1** | **0.2** | **0.3** | **0.4** |
| --- | --- | --- | --- | --- |
| **All patients (28%)** | 36 | 18 | 12 | 9 |
| **Low risk (18%)** | 55 | 28 | 19 | 14 |
| **High risk (43%)** | 23 | 12 | 8 | 6 |

**Table S13:** **Modelling of number-needed-to-treat to prevent 1 infection in patients categorized into high or low risk using the approach set out in the ‘modelling of clinical use’ section of the main manuscript**.

Figures are based on sampling patient at study day 2-3 (day 3-5 after ICU admission). The relative risk reductions are for hypothetical immunomodulatory agents. For reference, the on-going GRID trial of GM-CSF in critically ill patients (NCT02361528) , is powered on an anticipated relative risk reduction of 0.3 on a baseline 40% incidence of secondary infection.

| **Relative risk reduction (prevalence of subsequent infection)** | **0.1** | **0.2** | **0.3** | **0.4** |
| --- | --- | --- | --- | --- |
| **All patients (28%)** | 7818 | 1886 | 806 | 434 |
| **Low risk (18%)** | 13736 | 3288 | 1394 | 746 |
| **High risk (43%)** | 4102 | 1006 | 436 | 238 |

**Table S14:** **Modelling of sample size for trials of hypothetical immunomodulatory agents, based on sampling patient at study day 2-4 (day 3-5 after ICU admission).**

Calculations for a total study sample size with 80% power to detect a change in proportion developing infection with an alpha of 0.05, using a two-sided test for difference in proportions.

**Supplemental section references**

E1 Suetens C, Morales I, Savey A, et al. European surveillance of ICU-acquired infections (HELICS-ICU): methods and main results. *J Hosp Infect*. 2007;65 Suppl 2:171-173.

E2 Conway Morris A, Anderson N, Brittan M, et al. Combined dysfunctions of immune cells predict nosocomial infection in critically ill patients. *Br J Anaes*. 2013;111:778-787.

E3 Vincent J-L. Nosocomial infections in adult intensive-care units. *Lancet*. 2003;361:2068-2077.

E4 Groll D, To T, Bombardier C, Wright J. The development of a comorbidity index with physical function as the outcome. *J Clin epidem*. 2005;58:595-602.

E5 Vincent JL, Moreno R, Takala J, et al. The SOFA (Sepsis-related Organ Failure Assessment) score to describe organ dysfunction/failure. *Intensive Care Med*.1996:707-710.

**Supplemental Figure legends**

**Figure S1: Recruitment diagram**

**Figure S2**: **Summary of the 3 primary measures over time, relative to time of infection/to day 8 for patients not developing infection.**

Data shown as median and interquartile range for all samples available at each time point.

1. expression of CD88 by neutrophils.
2. expression of HLA-DR by monocytes.
3. T_regs_ as a % of all CD4+ lymphocytes.

**Figure S3: Panel A flow diagram of modelling of the proposed testing strategy at study day 0 (enrolment).**

**Panel B flow diagram of modelling of the proposed testing strategy study day 2-4.**
